# A Rare Case of Uterine Myxoid Leiomyosarcoma

**DOI:** 10.7759/cureus.44303

**Published:** 2023-08-29

**Authors:** Rashmi Wankhade, Anita Sajjanar, Pratibha Dawande, Obaid Noman

**Affiliations:** 1 Pathology, Datta Meghe Medical College, Datta Meghe Institute of Higher Education and Research, Nagpur, IND; 2 Pathology, Jawaharlal Nehru Medical College, Datta Meghe Institute of Higher Education and Research, Wardha, IND

**Keywords:** histopathology, uterine sarcomas, uterus, myxoid, leiomyosarcoma

## Abstract

A 49-year-old female patient was referred to a tertiary care hospital with a history of postmenopausal bleeding and abdominal pain that had persisted for the last two months. An abdominal examination revealed a huge mass that had been present for 12 weeks. A fibroid uterus was suggested by USG. A biopsy was done and sent to histopathology which revealed myxoid leiomyoma. Subsequently, the patient underwent total abdominal hysterectomy without bilateral salpingo-oophorectomy. Histopathological examination confirmed the diagnosis of myxoid leiomyosarcoma (MLMS) of the uterus.

## Introduction

Less than 1% of malignant female genital tract tumors are uterine sarcomas, which account for roughly 3% of all uterine tumors. The four most prevalent uterine sarcomas are carcinosarcoma, leiomyosarcoma (LMS), endometrial stromal sarcoma, and adenosarcoma, listed in decreasing frequency [1]. A palpable pelvic mass (54%), per vaginal bleeding (56%), and pelvic pain (22%), among other symptoms, are some of the presentations [2]. Myxoid LMS (MLMS) is a tumor variant of the uterine LMS variant, extremely rare with an annual incidence of 0.64 per one lakh women. It is exceedingly challenging to identify because of the aggressive disease course, clinical and histological heterogeneity, and ostensibly benign and bland cellularity [3]. King et al. provided the first detailed account of myxoid uterine LMS in 1982 [4]. MLMS are tumors that include myxoid stroma in at least 60% of the tumor area, have undergone microscopic examination, and exhibit light microscopy and immunohistochemical evidence of smooth muscle differentiation [5]. Correct diagnosis of MLMS is essential since, unlike other cancers, mitotic figures and abundant cellularity are not usual. Despite having a low mitotic index, these tumors have a startling myxoid look and behave very malignantly [6]. This case study describes a 49-year-old multiparous lady who had a total abdominal hysterectomy.

## Case presentation

A 49-year-old female patient presented with complaints of postmenopausal bleeding for the last two months associated with abdominal and pelvic pain. Blood tests performed prior to admission revealed the following results: hemoglobin 10.8 g/dl, iron 31 g/dl, red blood cells 4.280,000/L, white blood cells 7.400/L, and platelets 243.000/L. Cancer antigen (CA) 125, serum lactate dehydrogenase, and CA 19-9 were in the normal ranges.

On examination, the patient had a clinically pale appearance, but her vital signs were stable. A lump was felt in the mesogastric and hypogastric areas during an abdominal examination. It reached the umbilicus and had a firm consistency. The uterus measured 18 weeks in size and had a lump that was palpable on the anterior wall during a vaginal examination. Bilateral ovaries and fallopian tubes were both healthy. Trans-abdominal ultrasound examination revealed an increased size of the uterus (sagittal diameter: 55 mm, transverse: 107 mm, and longitudinal: 180 mm), an endometrial thickness of 2 mm, and a large fibroid localized to the myometrium. A provisional diagnosis of fibroid uterus was given, and a biopsy was sent to the histopathology department. Histopathological examination of the biopsy specimen revealed leiomyoma with myxoid changes as no associated atypia, and no mitotic figures could be identified. The patient next underwent a total abdominal hysterectomy without bilateral salpingo-oophorectomy. During surgery, a large fibroid measuring 4.8 x 3.5 x 2.6 cm was found. It lateralized to the isthmus and broad ligament. Ovaries and fallopian tubes on both sides were in good condition. In our surgical pathology section, the resected specimen was received for histopathological analysis.

**Figure 1:**
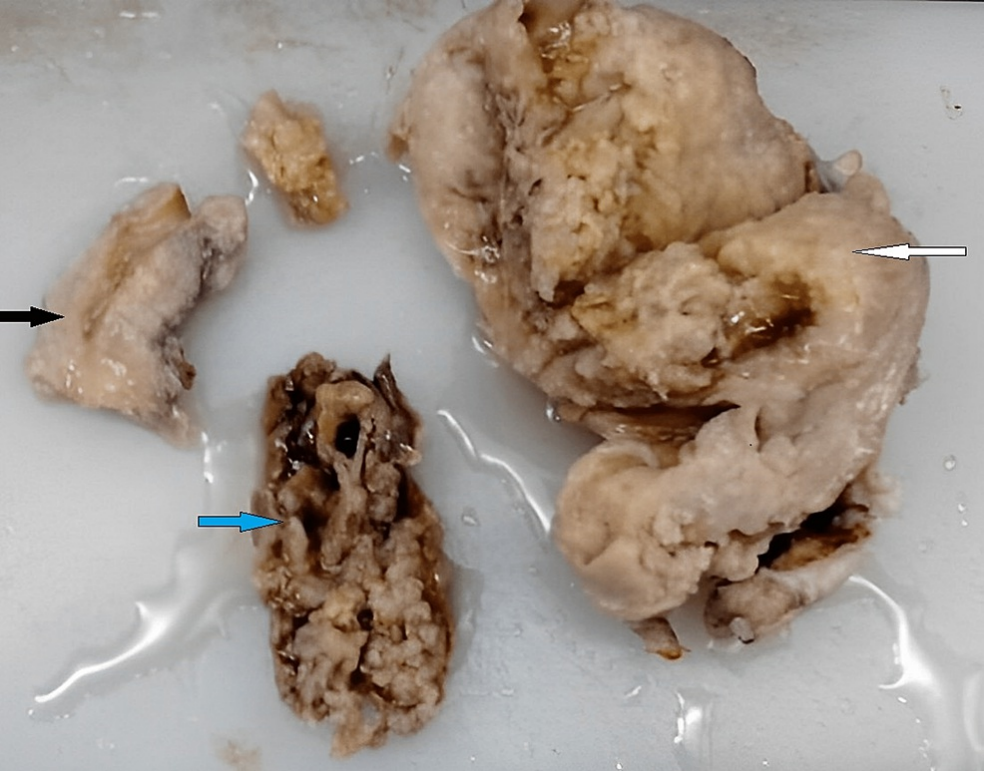
Distorted uterus with cervix and separate tissue mass The white arrow shows a distorted uterus, the black arrow shows a distorted cervix, and the blue arrow shows a separate tissue mass

The specimen received consisted of a distorted uterus with a separate piece of the cervix and a separate tissue mass. Distorted uterus with cervix measured 11.2 x 10.4 x 5.6 cm. Separate cervix measured 3 x 3 x 2 cm. A tissue mass that was sent separately was 4.8 x 3.5 x 2.6 cm in size. Endometrial thickness and myometrial thickness were 0.2 cm and 3 cm, respectively. The external surface of the uterus was bulky. On the cut section, the endometrial cavity was patent, and the myometrium showed a fleshy mass with grey-white to yellowish areas. The external surface of the separately sent tissue mass was grey-brown and congested. The cut section of the separately sent tissue mass was grey-brown, fleshy, gelatinous, and friable. Areas of necrosis were also seen (Figure 1).

**Figure 2:**
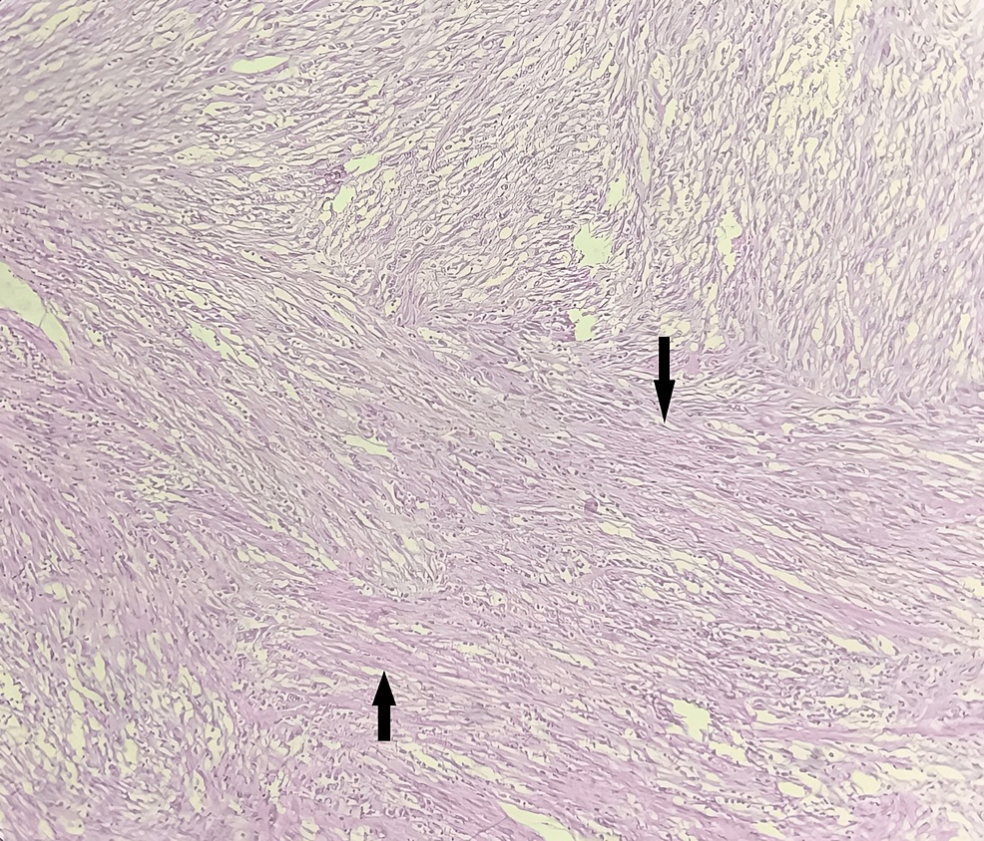
H&E-stained photomicrograph of the uterine tumor section showing highly cellular fascicles of spindle cells (low power view) The black arrows show highly cellular fascicles of spindle cells

**Figure 3:**
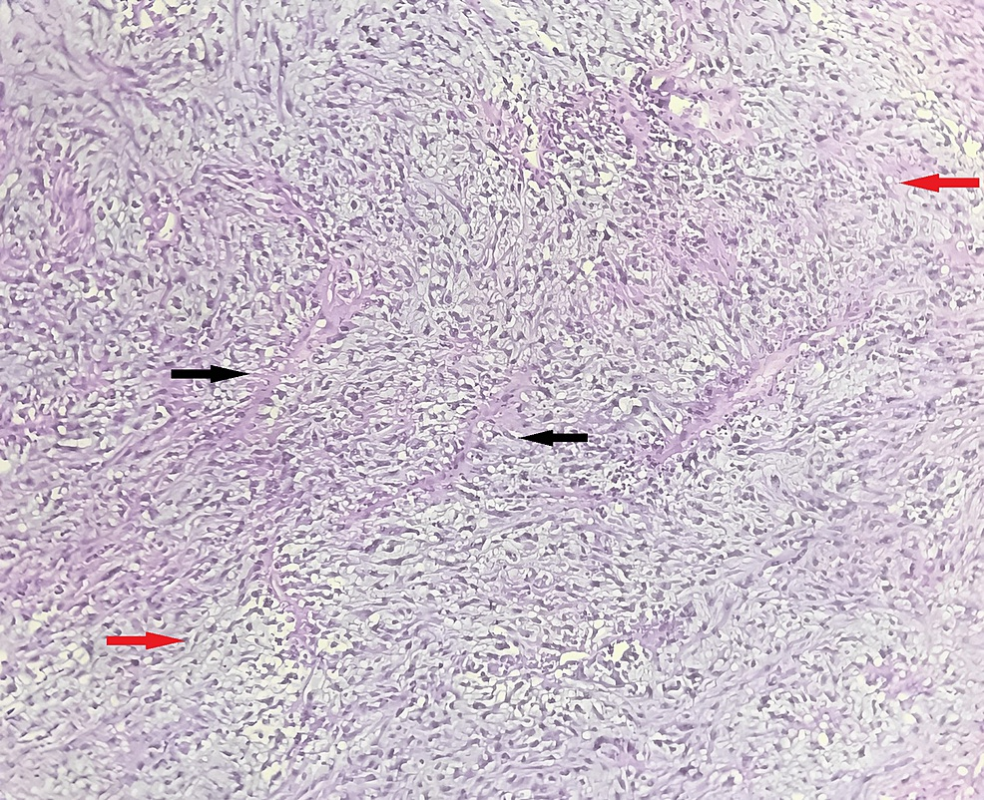
H&E-stained photomicrograph of the uterine tumor section showing tumor cells arranged in the form of fascicles with abundant myxoid stroma (medium power view) The black arrows show tumor cells arranged in the form of fascicles and the red arrows show myxoid stroma

**Figure 4:**
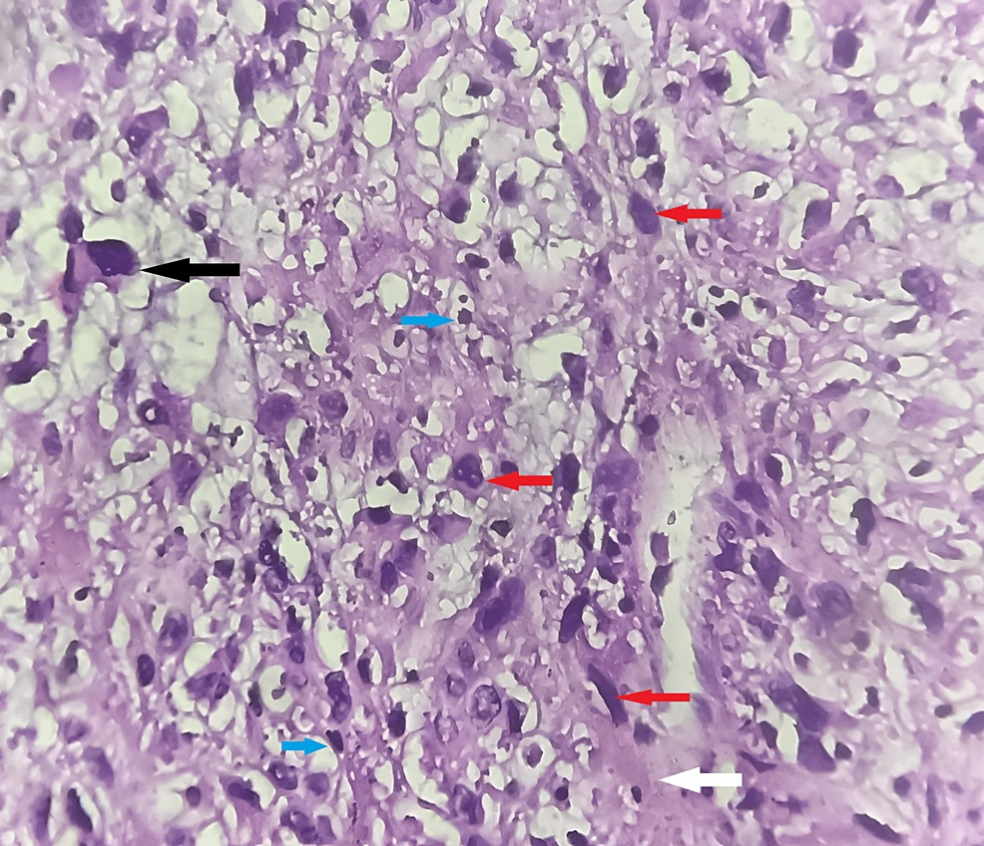
H&E-stained photomicrograph of the uterine tumor section showing atypical spindle cells, pleomorphic nuclei, and mitotic figures (high power view) The red arrows show atypical spindle cells with nuclear pleomorphism, the black arrow shows the bizarre cell, the blue arrow shows mitotic figures, and the white arrow shows myxoid stroma

On microscopic examination, the section studied from the endometrium revealed endometrial glands in the secretory phase with no evidence of atypia or malignancy. Sections from the cervix revealed that the ectocervix was lined by stratified squamous epithelium, and the endocervix was lined by columnar epithelium. The sub epithelium revealed lymphoplasmacytic infiltrate suggestive of chronic non-specific cervicitis. Sections taken from the myometrium and separate tissue mass were examined, and they revealed pleomorphic spindle cells with elongated nuclei, high-grade cytological atypia, eosinophilic cytoplasm, and a high rate of mitosis (12 mitotic figures per 10 high-power fields). They were arranged in fascicles, interlacing patterns, sheets, and nests. Few multinucleated giant cells were seen. Stroma showed myxoid changes. Areas of coagulative necrosis were also noted. Lymphovascular invasion was absent (Figures 2-4). Histopathological features were suggestive of MLMS of the uterus.

## Discussion

A proliferation of malignant smooth muscle cells with a lot of myxoid matrix is known as MLMS. Low cellularity and low mitotic index serve as distinguishing characteristics [7]. A very rare histological form of LMS is MLMS. Only 15% of women with LMS are under the age of 40, with ages 50 to 55 being the median [8]. Clinical symptoms may include an abdominal lump, abnormal vaginal bleeding, or bowel and bladder problems brought on by pressure. LMS is an aggressive cancer with a poor prognosis and a propensity for recurrence [9].

It is challenging to establish the preoperative diagnosis of uterine LMS. Vardi and Tovell [10] and Gallup and Corday [11] employed uterine curettage to diagnose uterine LMS in five of the 24 patients (20.8%) and in two of the eight patients (25%), respectively. A total abdominal hysterectomy with or without bilateral salpingo-oophorectomy is performed as the first therapeutic step when surgery is an option. Depending on the patient's biological characteristics, tumor removal or reduction might potentially be added to radiotherapy or chemotherapy [12]. According to King et al., the low mitotic index and high quantities of intercellular myxoid tissue in MLMS may prevent it from responding to chemotherapy or radiotherapy. As a result, it is unknown if adjuvant therapy works to prevent recurrence [13]. Although serum levels of CA125 in MLMS have been studied in several research, their significance has not been fully established [14,15].

The majority of research that has been published concurs that the number of mitoses per 10 high-power fields is a helpful diagnostic and prognostic factor in LMS. Burch and Tavassoli [16] claimed that studies on the mitotic index in MLMS have shown that at least 1/10 high power fields or 2/10 high power fields must be present, albeit it is extremely rare for a high mitotic index to be >57/50 high power fields. Additionally, tumor margins were invasive in nearly 30% of MLMS cases, and vascular invasion was prevalent. All of these factors highlight the tumor's capacity for infiltration and spread, raising the malignant potential of MLMS [17]. It is challenging to distinguish between traditional LMS and MLMS due to the rarity of the primary malignancy criteria (nuclear atypia, prominent mitotic figures, and hypercellularity). As a result, the majority of cases are determined by retrospective diagnosis [18].

Our patient was a postmenopausal woman who was experiencing unusual vaginal bleeding. With minimal opportunity for error, the first tissue diagnosis of myxoid leiomyoma was given which was based on clear-cut microscopic results of histopathological examination of the initial biopsy specimen. Despite the experience in diagnosis, it is unclear if the sarcomatous change took place later or if the initial microscopic look was deceiving. The absence of cellular atypia in the biopsy specimen must be emphasized, though. This example has been reported to emphasize the need to distinguish MLMS from a uterine leiomyoma with myxomatous alterations. There have been reports of an incidence of up to 15% for myxoid degeneration in benign leiomyomas, while myxoid transformation in LMS is extremely uncommon [19]. Due to the rarity of MLMS, it is challenging to reach a complete consensus on the diagnostic standards, which makes the diagnostic conundrum even more difficult to solve. Later on, with the histopathological examination of the total abdominal hysterectomy specimen, MLMS was the straightforward diagnosis.

## Conclusions

We reported a case of MLMS of the uterus. Menopausal state, pelvic symptoms, and the gross morphology of the tumor with fleshy, soft features all supported a malignant pathology. The uneven myometrial invasion, infrequent mitosis, cell pleomorphism, necrosis, and predominant myxoid component on histopathology provided the framework for the diagnosis of MLMS. On histopathological examination, we confirmed the diagnosis of MLMS of the uterus. Our experience supports the need for prudence in indicated clinical contexts and the requirement to be alert to the possibility of MLMS in such situations.
